# Endoscopic “Two-Step Method” for resection of a large tumor at the major duodenal papilla: A case report (with video)

**DOI:** 10.1097/MD.0000000000050721

**Published:** 2026-09-18

**Authors:** Dan Wu, DaYong Sun, ChaoHui Niu, Hong Wei

**Affiliations:** a Department of Gastroenterology, The Second People’s Hospital of Shenzhen City(The First Affiliated Hospital of Shenzhen University), Shenzhen, China.

**Keywords:** case report, endoscopic papillectomy, giant tumor of the major duodenal papilla, piecemeal resection, two-step method

## Abstract

**Rationale:**

Endoscopic papillectomy is the standard treatment for noninvasive tumors of the ampulla of Vater. However, reports on the endoscopic resection of large tumors at the major duodenal papilla are rare. The “two-step” endoscopic approach described herein represents the first such report for a giant lesion.

**Patient concerns:**

A 75-year-old female patient was admitted to the hospital with a 6-month history of abdominal distension. Gastroscopy revealed a giant tumor in the descending duodenum that prevented clear visualization of its full extent.

**Diagnoses:**

Duodenoscopy revealed a laterally spreading tumor, granular homogeneous type (LST-P, Paris 0-IIa), measuring approximately 60 × 50 mm, surrounding the major duodenal papilla. Contrast-enhanced computed tomography showed a 65 × 35 mm intraluminal lesion with no lymphadenopathy. Endoscopic ultrasonography confirmed mucosal and submucosal origin without biliary or pancreatic duct invasion. Biopsy revealed low-grade intraepithelial neoplasia. The final diagnosis was a giant laterally spreading tumor of the major duodenal papilla.

**Interventions:**

Endoscopic resection was performed using a novel “Two-Step Method.” In the first session, the bulk of the tumor was resected piecemeal to improve the operative field. Five days later, the residual lesion was resected by endoscopic mucosal resection after submucosal injection. Prophylactic biliary and pancreatic duct stents were placed upon completion of the second session.

**Outcomes:**

The patient commenced a cold liquid diet on the second postoperative day. She experienced no abdominal pain, hematochezia, or fever. Histopathology revealed a villous-tubular adenoma with low-grade intraepithelial neoplasia. No adenomatous tissue was identified at the cauterized margins of the submitted specimens. No complications occurred, and no recurrence was observed during the 12-month follow-up.

**Lessons:**

Few reports describe the endoscopic treatment of large duodenal tumors. This case demonstrates that the two-step method is a safe and effective alternative for giant duodenal mucosal lesions when single-session resection is precluded by limited operative space. Further experience is needed to establish consensus on the optimal management of such lesions.

## 1. Introduction

The ampulla was first described by Vesalius in 1543 and was later named after Abraham Vater. It is formed by the confluence of the common bile and pancreatic ducts.^[1]^ Ampullary tumors are relatively rare, with an annual incidence of <1 per 100,000 people, accounting for 0.6–0.8% of all gastrointestinal malignancies.^[2]^ Adenomas and adenocarcinomas of the major duodenal papilla account for ≥ 90% of all ampullary tumors. Duodenal papillary adenocarcinoma is a highly malignant and invasive tumor of the digestive system with a 5-year survival rate of only approximately 60%.^[3]^ Adenomas of the major duodenal papilla are likely to follow adenoma-to-carcinoma progression similar to that of colorectal adenocarcinoma.^[4]^ Although defined as benign, adenomas of the major duodenal papilla are considered precancerous lesions; therefore, complete resection is crucial for radical treatment of this disease.^[5]^ The symptoms of adenomas of the major duodenal papilla may not be obvious; if detected early, the tumors can be radically resected.^[6]^ Historically, adenomas of the major duodenal papilla have been surgically resected via local duodenectomy or pancreaticoduodenectomy.^[7,8]^ However, radical surgical procedures cause severe surgical trauma, have high mortality rates, and can lead to various complications. Local resection of small lesions is excessively traumatic.^[9]^ Therefore, ESP has become the preferred treatment method for papillary adenomas owing to its low degree of invasiveness and good safety.^[8]^ Most adenomas of the major duodenal papilla are single and sessile, with a diameter of 2 cm, and can be completely endoscopically resected by snare resection, which involves removing the mucosal layer of the duodenal wall around the major papilla, including the tissues around the openings of the bile duct and pancreatic duct.^[10]^ There are few reports in the literature regarding the selection of treatment methods for adenomas of the major duodenal papilla that are larger than 2 cm, and whether surgical intervention is required remains to be explored. This study describes the case of a 75-year-old female patient with a giant neoplastic lesion in the descending duodenum surrounding the major duodenal papilla. Endoscopic resection of lesions at this site is difficult and requires extensive experience in duodenoscopy. We successfully resected the lesion using the “two-step” method, and the patient had a good diagnosis at one-year follow-up. These findings suggest that this method is safe and effective for the treatment of giant duodenal mucosal lesions.

## 2. Case report

**Figure 1.**
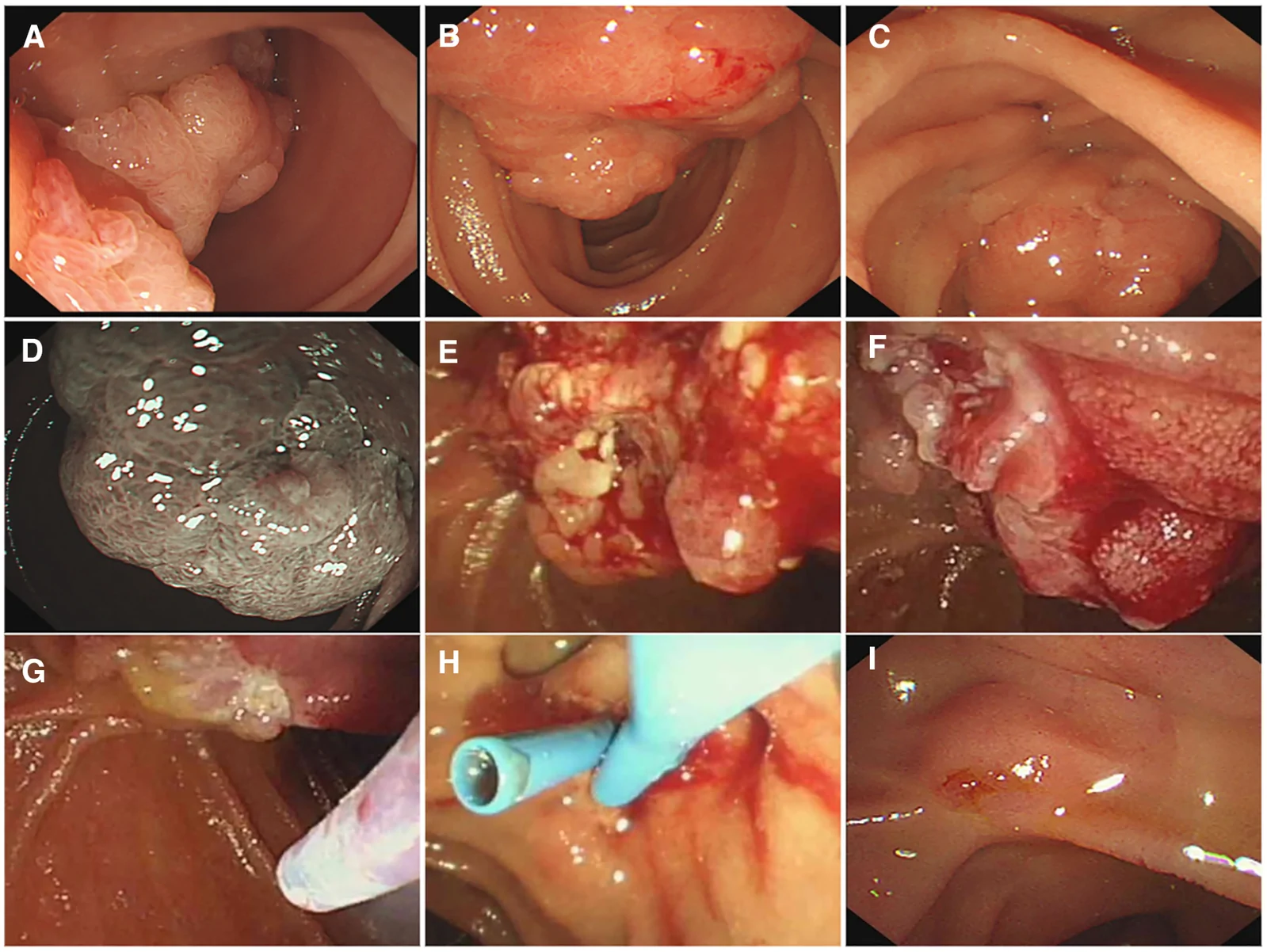
Side-viewing endoscope view of a tumor of the major duodenal papilla. (A) Frontal view. (B) Oral-side view. (C) Anal-side view. (D) Narrow-band imaging. (E) Piecemeal resection of part of the tumor. (F) Complete resection of the residual tumor by EMR. (G) Electrocoagulation for wound surface management. (H) Biliary and pancreatic duct stents were placed. (I) Scar formation in the duodenal papillary region.

**Figure 2.**
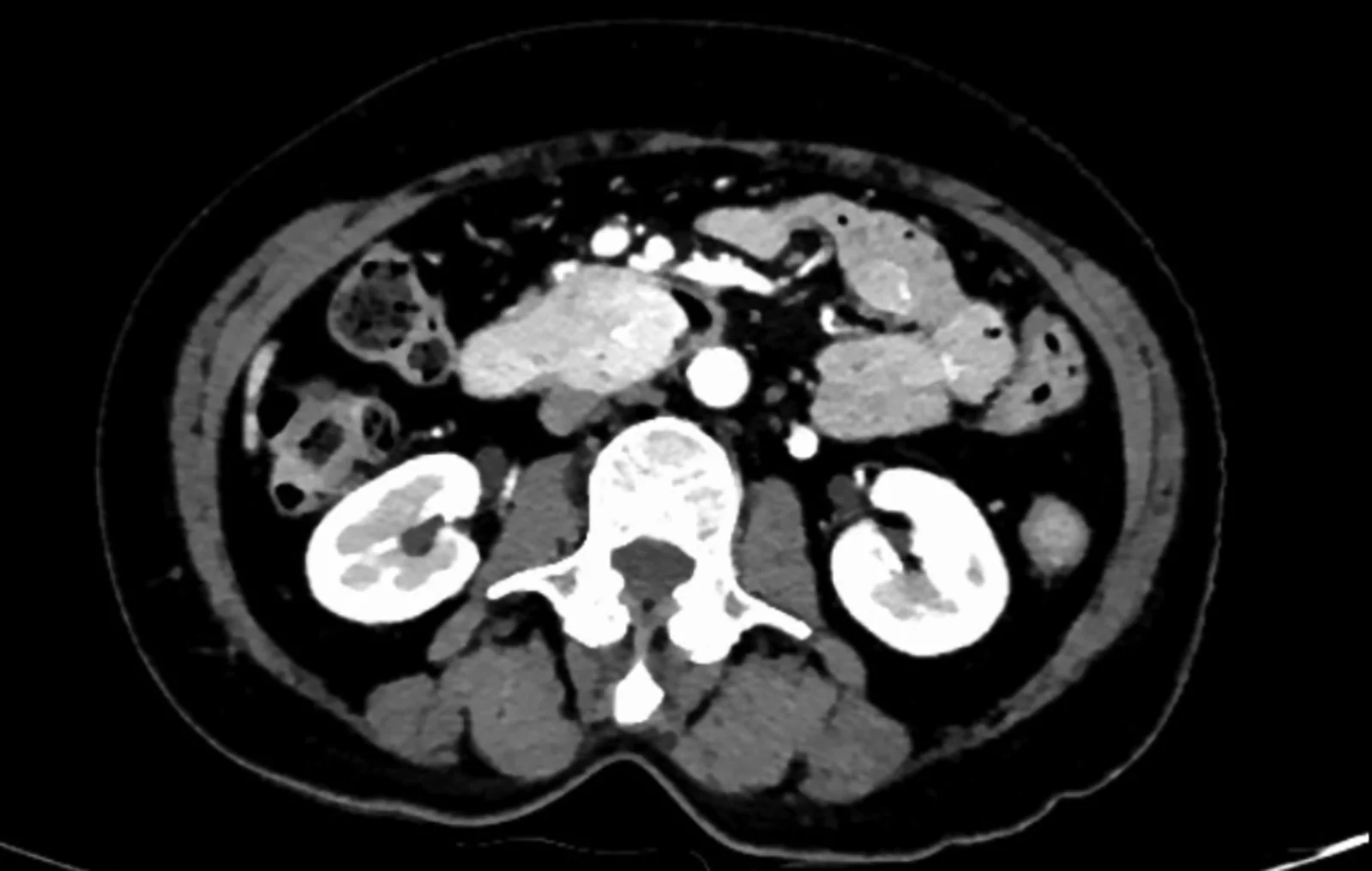
Abdominal enhanced CT shows a space-occupying lesion at the descending and horizontal parts of the duodenum; the lesion exhibits homogeneous enhancement on enhancement scan, with no enlarged lymph nodes in the periphery.

**Figure 3.**
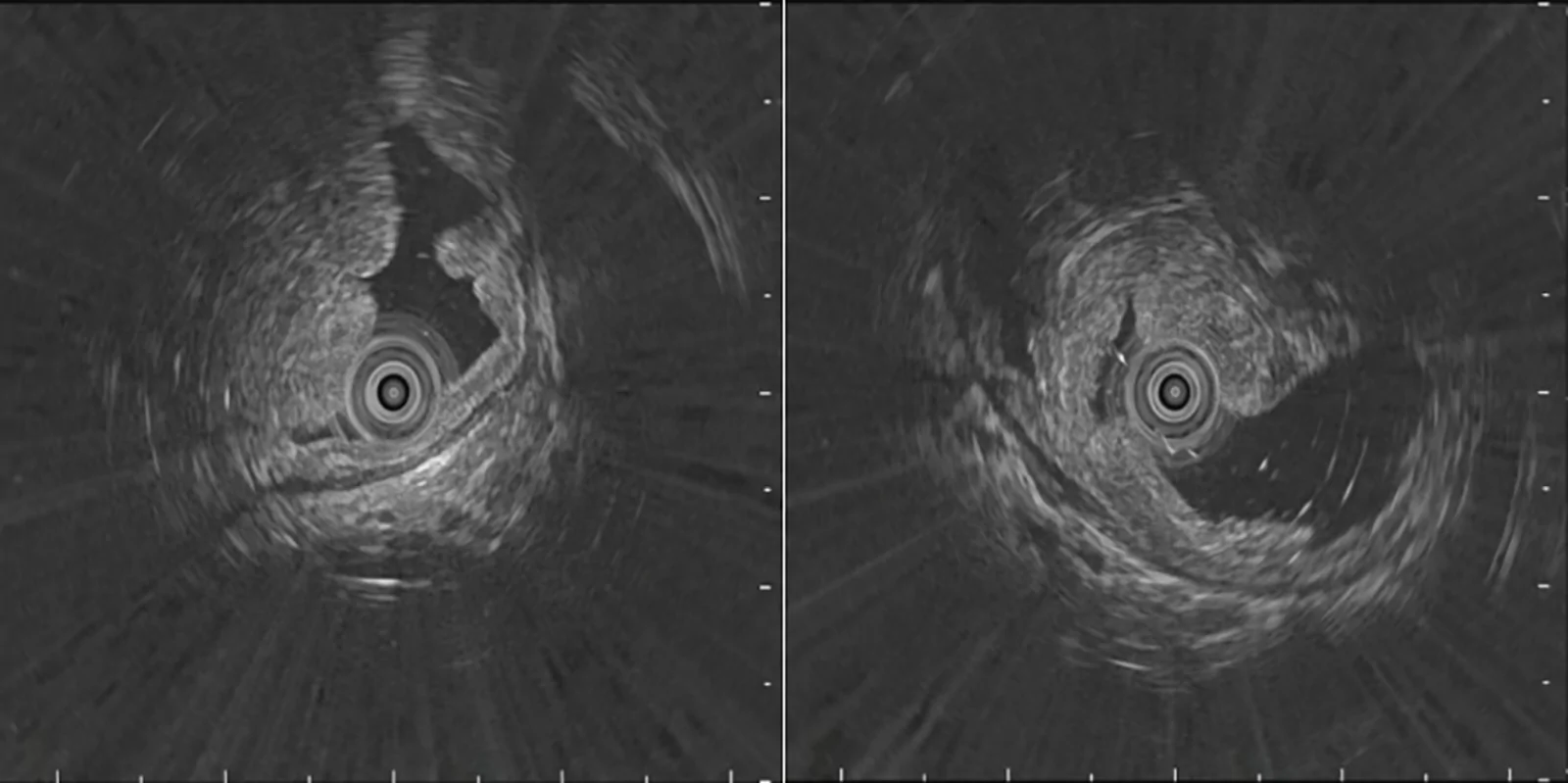
Endoscopic ultrasonography (EUS) suggests that the elevated lesion of the duodenum originates from the mucosal layer and invades the submucosal layer, with no invasion of the biliary and pancreatic ducts.

A 75-year-old female patient was admitted to the hospital because of “abdominal distension for half a year.” She had a history of hypertension, and her blood pressure was well-controlled. She denied smoking, alcohol consumption, or any other relevant habits. Routine blood tests, liver function, renal function, erythrocyte sedimentation rate, amylase levels, lipase levels, and a full set of tumor marker levels were all within normal limits. Gastroscopy revealed a giant tumor in the descending duodenum, making it impossible to observe the entire tumor clearly. Duodenoscopy revealed a giant mucosal protrusion in the descending duodenum, measuring approximately 60 × 50 mm, surrounding the major duodenal papilla, with a partially lobulated surface. Narrow-Band Imaging revealed relatively normal microstructures and regular microvascular and microsurface structures. According to the Paris classification of superficial lesions, it was classified as LST-P, Paris classification 0-IIa (Fig. 1). Contrast-enhanced CT of the entire abdomen revealed thickening of the intestinal wall at the horizontal segment of the descending duodenum, with a soft tissue density shadow protruding into the intestinal lumen, measuring approximately 65 × 35 mm with ill-defined boundaries. An enhancement scan revealed a slightly uneven and obvious enhancement, and no adjacent enlarged lymph nodes were found (Fig. 2). EUS revealed that the protruding lesion of the duodenum originated from the mucosal and submucosal layers, presenting as a moderate to high echo. The confluence structure of the bile and pancreatic ducts was normal, with no evidence of lesion invasion (Fig. 3). Pathological results of the tumor biopsy indicated low-grade intraepithelial neoplasia. On the basis of these findings, we determined that the tumor was limited to the mucosal layer of the major papilla, which is a good indication for EMR. However, the tumor was large, the operative space was limited, and endoscopic resection was difficult. After a full departmental discussion, considering that the lesion was benign and that the patient was elderly, we decided to attempt minimally invasive endoscopic resection. The operation was performed by Chief Physician Dayong Sun, who has extensive experience in our department. Given the large size of the tumor, the operation was performed in 2 stages, i.e.., the “two-step” method. In the first stage, most tumors were resected into pieces using an endoscopic snare. After resection, the field of view was clear, the operating space improved, and the specimen was sent for pathological examination. The surgery was successful (see Supplementary Video 1, Supplemental Digital Content 1). Five days later, the remaining tumor was resected using EMR. For the remaining lesion, indigo carmine was first injected submucosally, and the lifting sign was positive. The muscularis propria was separated and the fully lifted lesion was resected using an electric snare (see Supplementary Video 2, Supplemental Digital Content 2). EMR was used to completely resect the remaining tumor and the normal mucosa around the tumor to ensure negative resection margins. After the operation, there were no residual lesions, bleeding, or perforations at the wound site. Under radiographic guidance, biliary and pancreatic duct stents were placed to prevent pancreatitis and cholangitis. The surgery was successful. All resected fragments were retrieved intact and submitted separately in sequentially labeled containers according to the order of resection. We acknowledge that precise anatomical orientation (oral vs anal side) of individual fragments could not be reliably determined after piecemeal retrieval. The pathologist examined each fragment for cauterized margins and mapped the overall distribution of adenomatous tissue across all submitted specimens. The absence of adenomatous tissue at the cauterized edges, combined with the 12-month endoscopic follow-up showing no residual or recurrent lesion, supports a conclusion of “clinical complete resection” rather than a definitive pathological R0 status.The final pathological result revealed a villous-tubular adenoma with low-grade intraepithelial neoplasia. No adenomatous tissue was identified at the cauterized horizontal or vertical margins of the submitted specimens.Amylase and lipase levels, routine blood test parameters, C-reactive protein (CRP) levels, and liver function were rechecked 6 hours and the day after the operation, and no obvious abnormalities were detected. The patient was then started on a cold liquid diet. Five days after the operation, the patient had no discomfort such as abdominal pain, hematemesis, or hematochezia, and was discharged from the hospital as planned. Duodenoscopy reexaminations at 3, 6, and 12 months postoperatively revealed no signs of recurrence, and continuous monitoring is ongoing.

## 3. Discussion

Tumors of the major duodenal papilla are uncommon, accounting for approximately 5% of all gastrointestinal tumors. Pancreaticoduodenectomy was regarded as the standard treatment for adenomas of the major duodenal papilla more than 20 years ago. In 1983, ESP was first reported in patients with adenomas of the major duodenal papilla.^[11]^ After years of medical development, endoscopic surgery has been found to be a safe and minimally invasive alternative to pancreaticoduodenectomy in the treatment of adenomas of the major duodenal papilla. Most literature focuses on endoscopic treatment for lesions with diameters <2 cm, whereas reports on endoscopic treatment for giant adenomas of the major duodenal papilla are rare.^[12]^ In accordance with the Paris classification system for large sessile or flat neoplastic lesions of the digestive tract, lesions with diameters >10 mm, low vertical axes, and lateral extensions along the inner wall of the lumen are defined as LSTs.^[13,14]^ Many studies have suggested surgical resection for larger lesions (>30 mm) and for those with obvious extrapapillary extensions.^[15–17]^ Although surgery is a treatment option for patients with adenomas of the major duodenal papilla, a notable result from a series of studies on LST resection mainly in elderly patients is that compared with the expected median hospital stay of 5 days for local surgical resection and up to 3 weeks for radical surgery, the reported overnight hospital stay of the patients was shorter (median 1 night).^[18]^ Therefore, minimally invasive endoscopic resection remains the preferred treatment method for large tumors of the major duodenal papilla (diameter > 30 mm).

Biopsy before papillectomy is effective for determining the appropriate treatment plan for patients. The American Society for Gastrointestinal Endoscopy (ASGE) recommends obtaining biopsy specimens from all suspected ampullary neoplastic lesions before endoscopic resection.^[19]^ However, the unpredictability of biopsy results highlights the importance of comprehensive examination and complete resection for accurate histopathological examination, which is also supported by expert opinion.^[20]^ To observe the papilla and evaluate the feasibility of endoscopic resection, EUS, which is superior to CT or MRI, can be used to assess the depth of the tumor and presence of intraductal growth into the bile duct or pancreatic duct. A meta-analysis revealed that the pooled sensitivity and specificity of EUS for diagnosing T1-stage ampullary tumors were 77% and 78%, respectively. However, CT or MRI is more helpful for detecting distant and lymph node metastases.^[21]^ In this case, the preoperative pathological result of duodenal papillary tumor biopsy indicated low-grade intraepithelial neoplasia, and neither EUS nor CT detected bile duct or pancreatic duct invasion. Although the patient had a giant adenoma of the major duodenal papilla and was an elderly woman (75 years old), after a full departmental discussion, considering previous relevant literature reports and factors including age, surgical treatment was abandoned and endoscopic treatment was ultimately chosen.

The selection of this surgery was challenging because the duodenal space was narrow, there were many adjacent surrounding organs, the risks of perforation and bleeding were high, and it was extremely difficult to completely resect the lesion. Very few reports on the endoscopic resection of giant adenomas of the major duodenal papilla have been published. Among these, only a single study described 10 cases of adenomas of the major duodenal papilla classified as LST-P (>30 mm) that were treated with endoscopic resection, and all operations were successful. However, a standardized single-EMR technique was used.^[18]^ This method elevates the lesion after EMR injection, which makes the operating space even narrower and the operation more difficult, especially for larger lesions (>50 mm) where there is no operating field of view. On the basis of the above reasons, we chose to complete the operation in 2 stages for this case, which was named the “two-step” method. First, a snare was used to resect the tumor in pieces to expose the root of the lesion and improve the operating space. In the second step, EMR was used to completely resect the remaining tumor and the normal mucosa around the tumor to ensure negative resection margins.

The rationale for the 5-day interval between the 2 procedures is multifactorial. First, the initial piecemeal resection reduced the tumor bulk, allowing the residual lesion to retract and the surrounding mucosa to partially recover, thereby improving the endoscopic field of view for the second session. Second, the interval allowed time for the initial pathological examination to confirm the benign nature of the lesion (low-grade intraepithelial neoplasia) and rule out invasive carcinoma before completing the definitive resection. Third, it minimized the risk of procedure-related complications (e.g., perforation, bleeding, and pancreatitis) by avoiding a prolonged single-session procedure in an elderly patient. Fourth, it allowed the endoscopist to reassess the anatomical landmarks and the exact extent of the residual lesion after the initial edema had subsided. Compared with single-session piecemeal resection of a 65 mm lesion, which carries a high risk of “lost field” due to massive submucosal injection and bleeding, the two-step approach converts a high-risk, uncontrolled procedure into two manageable, deliberate resections.

During the second session, mild inflammatory changes were present at the resection margins, but significant fibrosis had not yet developed. The lifting sign remained positive after submucosal injection of indigo carmine, indicating that the submucosal layer was still adequately separable from the muscularis propria. However, the technical challenges included: precise identification of the residual adenomatous tissue amid the granulation tissue at the wound base; careful preservation of the major duodenal papilla architecture to avoid ductal injury; and maintaining adequate submucosal elevation in a narrowed operative field while ensuring complete circumferential resection of the residual lesion with a sufficient safety margin.

Studies in the literature have reported that lesions larger than 2 cm can be resected into pieces, and that these operations can be successful. Interestingly, there was no difference in the residual rate between the 2 groups 3 months after the operation. One of the reasons may be that efforts were made in the resection attempt regardless of whether en bloc or piecemeal resection was used.^[22]^

To contextualize the safety benefits of our two-step approach, we compared our case with previously reported endoscopic resections of giant papillary adenomas (>50 mm). Hopper et al.^[18]^ reported the largest series of LST-P (>30 mm) treated with single-session EMR, achieving complete resection in all 10 cases; however, their technique required extensive submucosal injection, which risks “lost field” in lesions exceeding 50 mm. In contrast, Itoi et al^[23]^ and Van der Wiel et al^[24]^ reported that piecemeal resection of large ampullary lesions was associated with higher rates of residual adenomatous tissue and postoperative pancreatitis (11.1–58.3%). More recently, Wang et al^.[22]^ described a modified single-session papillectomy for large papillary tumors, but their approach still required prolonged procedural time (mean 87 minutes), increasing anesthesia-related risks in elderly patients. Our two-step method offers distinct advantages for giant lesions (>50 mm): by debulking the tumor in the first session, we restored the operative field and avoided the “lost field” phenomenon caused by excessive submucosal injection; the staged approach reduced single-session procedural time, minimizing cardiopulmonary stress in our 75-year-old patient; and pathological confirmation of benign histology between sessions allowed us to proceed with confidence, whereas single-session approaches lack this interim safety checkpoint. These comparative data support the hypothesis that the two-step method is particularly suited for giant LST-P lesions in elderly or high-risk patients where single-session resection would be technically hazardous.

The application of submucosal injection during endoscopic resection of adenomas of the major duodenal papilla has been controversial. For tumors originating from the papilla and growing into the duodenal mucosa or lesions located at the edge or inside the diverticula, this injection is very beneficial because these characteristics help lift the lesion and clear the field of view, thereby facilitating the use of a snare. Submucosal injections can reduce the incidence of perforation and complications after endoscopic resection and help determine the depth of invasion. Notably, the amount of submucosal injection fluid must be considered because the duodenal lumen is narrow and excessive mucosal elevation may affect the observation of the field of view and the use of the snare.^[7]^

In this case, the tumor was very large, and great effort was made to completely resect the lesion. Finally, the “two-step” endoscopic treatment method was chosen, with no residual lesions, bleeding, or perforation at the wound site, and no tumor recurrence observed within 1 year after the operation. Cohort studies have shown that after resection of adenomas of the major duodenal papilla, pancreatitis is the most common complication, followed by bleeding, perforation, stenosis, and cholangitis, with an overall incidence of 6.1–58.3%.^[23]^ Other studies have shown that the incidence of pancreatitis, papillary stenosis, and cholangitis is approximately 11.1%, 2.4%, and 4.5%.^[24,25]^ The placement of a pancreatic stent is intended to reduce the incidence of pancreatitis complications. Studies have shown that patients who do not receive pancreatic stents are more likely to develop pancreatitis after papillectomy.^[26]^ Prophylactic pancreatic duct and bile duct stent placement have been shown to effectively prevent pancreatitis and cholangitis, and are thus recommended according to many guidelines and expert consensuses.^[2,23,27]^ In this case, pancreatic duct and bile duct stents were placed after the completion of the second session to prevent pancreatitis and cholangitis, and no bleeding, perforation, pancreatitis, or cholangitis occurred. Some reports have suggested that for patients with complete adenoma resection, endoscopic monitoring should be performed at intervals of 6 months for at least 2 years.^[28]^ In this case, during the 12-month postoperative observation period, duodenoscopic monitoring was performed at 3, 6, and 12 months, and no tumor recurrence was detected. Monitoring is currently ongoing.

## 4. Conclusion

As a detailed review and in-depth analysis of a single case of endoscopic “two-step” resection of a giant tumor of the major duodenal papilla, this study has important value for theoretical exploration, clinical practice, and technical promotion of endoscopic diagnosis and treatment of digestive system tumors and provides a key reference for the diagnosis and treatment of such complex diseases. Currently, reports on the endoscopic treatment of giant duodenal tumors are rare, and the “two-step” endoscopic treatment method adopted in this case is the first such report. Of course, more experience in endoscopic treatment of such cases is needed to reach a consensus on the optimal treatment method for giant adenomas of the major duodenal papilla and to provide a core research direction for the subsequent development of “multicenter, large-sample” clinical studies.

## Acknowledgments

This article specifically acknowledges Director Sun Dayong for serving as chief surgeon for the entire procedure. We confirm that he has given permission to be named in this acknowledgment.

## Author contributions

**Conceptualization:** Dan Wu, DaYong Sun.

**Formal analysis:** Dan Wu.

**Investigation:** Dan Wu, Hong Wei.

**Methodology:** Dan Wu, ChaoHui Niu.

**Resources:** Dan Wu, ChaoHui Niu.

**Supervision:** Dan Wu, DaYong Sun.

**Validation:** Dan Wu, Hong Wei.

**Writing – original draft:** Dan Wu, DaYong Sun, ChaoHui Niu, Hong Wei.

**Writing – review & editing:** Dan Wu, DaYong Sun, ChaoHui Niu, Hong Wei.

**Project administration:** DaYong Sun.

**Software:** ChaoHui Niu.

**Data curation:** Hong Wei.

**Visualization:** Hong Wei.




